# An endometrium of type C along with an endometrial thickness of < 8 mm are risk factors for ectopic pregnancy after stimulated cycles with fresh embryo transfer

**DOI:** 10.1186/s12884-023-05920-y

**Published:** 2023-10-06

**Authors:** Ying Zhao, Aizhuang Xu, Dong’e Liu, Nenghui Liu, Yumei Li, Zhongyuan Yao, Fen Tian, Hongying Tang, Yanping Li

**Affiliations:** 1 Department of Reproductive Medicine, Xiangya Hospital, Central South University, Changsha, Hunan 410000 People’s Republic of China; 2 Hunan Provincial Key Laboratory of Regional Hereditary Birth Defects Prevention and Control, Changsha Hospital for Maternal & Child Health Care Affiliated to Hunan Normal University, Changsha, Hunan 410000 People’s Republic of China; 3 Clinical Research Center for Women’ s Reproductive Health in Hunan Province, Changsha, Hunan 410000 China

**Keywords:** Ectopic pregnancy, Endometrium type, Endometrial thickness, Stimulated cycles, Fresh embryo transfers

## Abstract

**Background:**

The study investigated whether specific ultrasonographically observed endometrial features (including endometrium type and thickness) were linked to ectopic pregnancy after stimulated cycles with fresh embryo transfer.

**Method:**

Of 6246 pregnancy cycles after fresh embryo transfer, 6076 resulted in intrauterine pregnancy and 170 in ectopic pregnancy. The primary outcome of the study was ectopic pregnancy, with the main variables being endometrium type and endometrial thickness. Univariate and subsequent multiple-stepwise logistic regression analyses were used to identify the risk factors of ectopic pregnancy.

**Results:**

1. Compared with patients with an endometrial thickness ≥ 8 mm, the adjusted odds ratio for those with an endometrial thickness < 8 mm was 3.368 (*P* < 0.001). The adjusted odds ratio for women with a type-C endometrium was 1.897 (*P* = 0.019) compared with non-type C. 2. A larger dose of gonadotropin used during controlled ovarian hyperstimulation was a protective factor against ectopic pregnancy (*P* = 0.008). 3. The GnRH antagonist protocol (*P* = 0.007) was a risk factor for ectopic pregnancy, compared with the use of GnRH agonists.

**Conclusion:**

(1) An endometrial thickness < 8 mm coupled with a type C endometrium significantly increased the risk of ectopic pregnancy after fresh embryo transfer. (2) A thin endometrial thickness and a type C endometrium could be further related to an abnormal endometrial receptivity/peristaltic wave. (3) Patients at a high risk of ectopic pregnancy should therefore be given special attention, with early diagnosis during the peri-transplantation period may assist in the prevention of ectopic pregnancy.

## Introduction

Ectopic pregnancy (EP) is an anomalous form of pregnancy whereby the embryo is implanted outside the uterine cavity [1]. In developing countries, 1% of maternal deaths can be attributed to this condition, with the figures rising to as high as 5% for developed countries [2]. Thus, EP can represent a significant social and economic burden.

Although Assisted Reproductive Technology (ART) should theoretically reduce the incidence of EP as the fallopian tubes are not involved in fertilization or embryo transfer (ET), it occurs in about 1–2% of spontaneous pregnancies [3] and up to 1.4–5.4% in ART [4, 5]. The risk factors of EP after ART include tubal factor infertility (TFI) [6–11], multiple embryos per transfer, [6] and fresh embryo transfer in stimulated cycles in comparison to thawed embryo transfer cycles [12–17].

During controlled ovarian stimulation (COS), ultrasound is routinely used to monitor the endometrial thickness (EMT) as well as the type of endometrium, with some studies suggesting that the thicker the EMT, the lower the incidence of EP [18–20]. However, to date, no studies have taken the endometrium type into account nor considered possible correlations between combined endometrial features and EP after ART. Furthermore, while previous studies were specifically focused on frozen embryo transfer cycles [18] or undertook a mixed analysis of both fresh and frozen embryo cycles [19, 20], none have analyzed fresh embryo transfers separately. Thus, the main aim of the current work was to investigate whether the endometrium type as well as EMT were related to EP occurrence, especially after stimulated cycles involving fresh embryo transfer.

## Methods

### Definition of clinical outcomes

The blood β-human chorionic gonadotropin (β-hCG) levels were examined 12 days after ET In this case, they were considered to be negative or positive when their β-hCG levels were respectively below 5 IU/L or above 15 IU/L. Some patients were also considered as indeterminate when their β-hCG amounts were between 5 and 15 IU/L but they were subsequently deemed to be positive if the levels increased after 48 h. All positive women underwent sonography 4–5 weeks after ET.

The possible outcomes encountered after ET included intrauterine pregnancy (IUP), indicating the presence of one or more intrauterine gestational sacs, and EP, defined as the presence of a gestational sac/mass outside the uterine cavity. In contrast, heterotopic pregnancy (HP) was diagnosed when intrauterine gestational sacs and ectopic pregnancy were observed simultaneously.

### Study design and patients

This study was performed in accordance with the ethical guidelines of the Declaration of Helsinki, revised in 1983. The participants were recruited from patients who had undergone stimulated cycles with fresh embryo transfer at the Reproductive Medicine Centre of Xiangya Hospital, Central South University, Changsha, China, between January 2014 and November 2021. Patients were examined by sonography at different stages of their menstrual cycles at least twice before the ART procedure. They were then further examined by hysteroscopy and treated when cavity abnormalities were observed.

Clinical information was acquired from the medical records. Patients with the following conditions were excluded: (1) cryopreserved embryos; (2) biochemical pregnancy; (3) heterotopic pregnancy; (4) cesarean scar pregnancy; (5) absence of endometrial data; (6) uterine anomaly; (7) donor oocytes cycles; (8) non-pregnant cycles.

EP was the main outcome examined while the main variables were endometrial type and EMT. Demographic data included age, parity, gravidity, etiology of infertility, infertility duration (years), ovarian stimulation protocol, number of embryo(s) per transfer, number of oocytes retrieved, duration of gonadotrophin (Gn) (days), total Gn dose (IU), method of insemination, basal FSH, and body mass index (BMI) as evaluated by the patient’s doctor in charge. Patients were diagnosed with tubal factor infertility (TFI) if any of the following was noted: hydrosalpinx, previous salpingectomy, previous EP, or tubal scarring, including occlusion. In addition, patients with any two of the following conditions were diagnosed with polycystic ovary syndrome (PCOS): clinical and/or biochemical hyperandrogenism, ovulatory dysfunction, and PCOM (polycystic ovary morphology). Finally, women were considered as presenting diminished ovarian reserve (DOR) if the results of their ovarian reserve test were abnormal (i.e., anti-Mullerian hormone (AMH) < 0.5–1.1 ng/ml or antral follicular count (AFC) < 5–7 follicles), or if any of the risk factors of poor ovarian response (POR) was observed. The follow-up rate in the study was 100%.

### Ultrasound measurement

EMT and endometrium type were assessed on the day of HCG injection (trigger day) by transvaginal 8 MHz ultrasonography with Doppler ultrasound (GE Voluson E10, USA). As described by Bredella et al. [21], the EMT was measured by transvaginal sonography, along a sagittal plane and with the maximal anteroposterior thickness used. The morphology of the endometrium was classified into one of three types: type A, with a trilaminar pattern (a triple-line pattern), consisting of outer, hyperechoic middle and hypoechoic inner layers as well as evident echoes at the intrauterine center line; type B, having a relatively homogeneous hyperechoic endometrium, with an obscure intrauterine center line echo and unclear endometrial layers but with clear interfaces between the endometrium and muscular layers; type C, having homogeneous hyperechoic endometrium without an intrauterine center-line echo.

### Procedure for inducing ovulation and IVF/ICSI-embryo transfer

The protocols selected for stimulation were tailored to each individual and have been described thoroughly in previous publications [22, 23]. Ovarian stimulation was initiated at the basal state of the ovaries [24] and was performed using human menopausal gonadotrophin (Lebaode) and/or exogenous highly purified follicle-stimulating hormone (FSH, Lishenbao). The starting dose of gonadotrophin (Gn), in this case, ranged from 112.5 to 300 IU, depending on the age of the patient, antral follicular counts (AFC), and basal FSH level (bFSH). HCG (6000-10 000 IU; Profasi; Serono, Italy) was then injected when at least two follicles reached ≥ 18 mm in diameter, with oocytes subsequently retrieved 36 h after the trigger. This was followed by routine in vitro fertilization (IVF)/ intracytoplasmic sperm injection (ICSI) before transferring a maximum of three high-quality embryos with a Cook catheter under transabdominal ultrasound guidance following the standard procedures of our center 72 h after oocyte retrieval. The luteal phase was eventually supported by vaginal micronized progesterone (Utrogestan) using a dose of 200 mg, three times a day, along with oral progesterone capsules (Qining) at a dose of 200 mg once a day, with both treatments simultaneously starting since the day of oocyte retrieval and lasting for 75 days.

### Statistics

Variables were selected based on previous studies [25–27] and data availability.

SPSS version 23 (IBM, USA) was used for data analysis. For quantitative data, results are presented as mean ± standard deviation (SD) or median (interquartile range) for normally and non-normally distributed data, respectively. Categorical data are presented as the number of cases (percentage).

Univariate analysis was performed using the Pearson Chi-square test for categorical variables, while two-sample t-tests and Mann-Whitney U tests were used where appropriate. Lastly, the independent risk factors for EP were identified based on stepwise multiple logistic regression analysis. Overall, differences were considered statistically significant at *P* < 0.05.

## Results

**Fig. 1:**
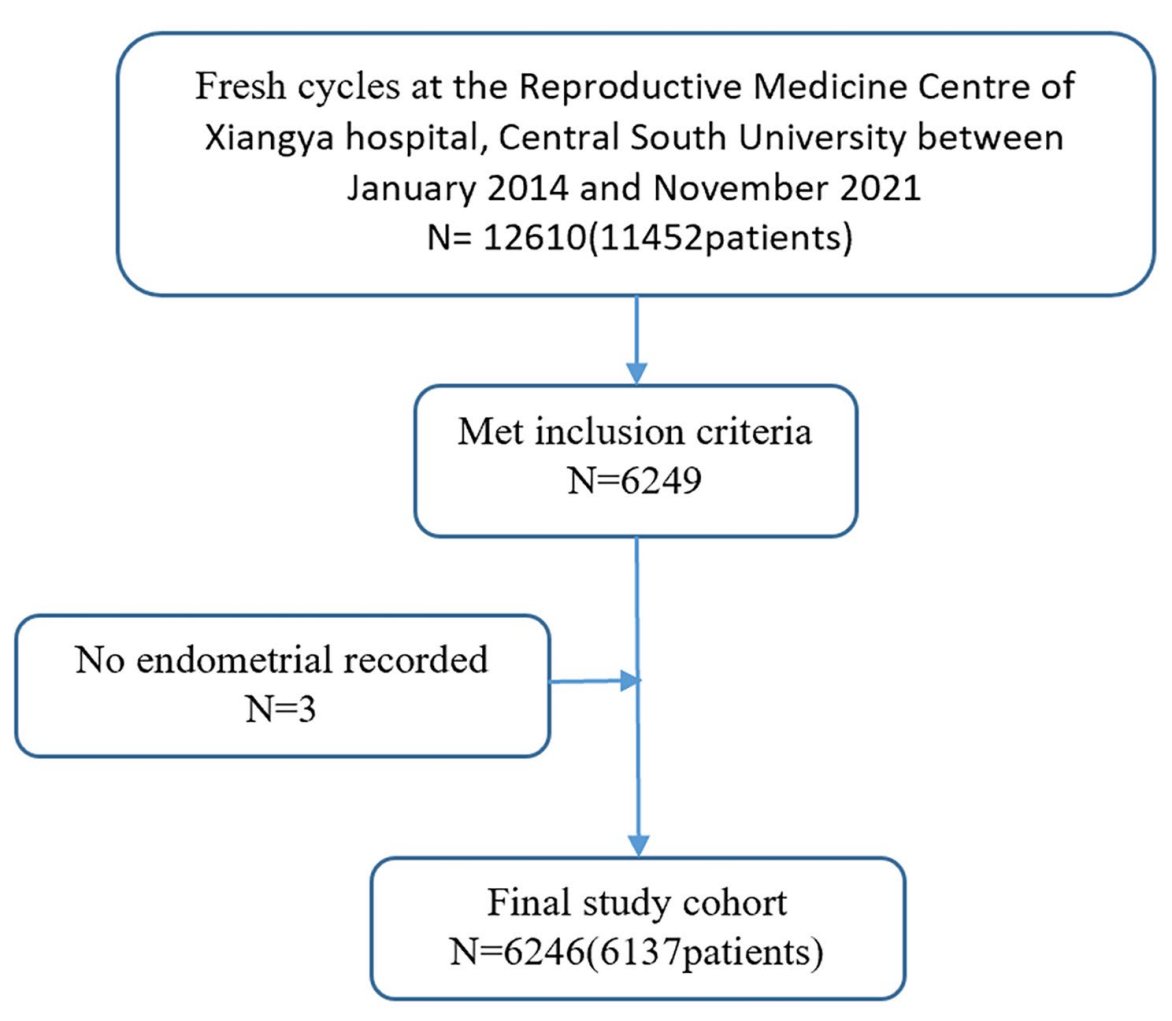
Flow chart of patients

The cycle selection process used to obtain the final study sample is shown in Fig. 1. The study included 6246 IVF cycles of which 6076 were IUP and 170 were EP cycles.

### Single-factor analysis

Table 1 shows the baseline characteristics of the patients together with the ovarian responses. There were no significant differences between patients in terms of BMI, age, previous history of ectopic pregnancy, infertility duration, type of infertility, gravidity, bFSH, duration of Gn, number of oocytes retrieved, and number of embryo(s) per transfer. However, significant differences were observed in terms of the method of insemination, total Gn dose, and ovarian stimulation protocol, as well as the presence or absence of TFI, male factor infertility, and PCOS (*P* < 0.05).

**Table 1:** The baseline characteristics of the patients

**Variable**	Intrauterine pregnancy, **n = 6076**	Ectopic pregnancy,**n = 170**	***P value***
**Age (years)**	**30(27,33)**	**30(27,33)**	**0.606**
**Body mass index (kg/m** ^**2**^ **)**	**21.6(19.83,23.8)**	**21.5(19.56,23.4)**	**0.445**
**Infertility type**			**0.284**
**Primary infertile**	**3148(51.8%)**	**81(47.7%)**	
**secondary sterility**	**2928(48.2%)**	**89(52.3%)**	
**Infertility duration (years)**	**3(2,5)**	**3(2,6)**	**0.450**
**Previous history of ectopic pregnancy**			**0.069**
**=0**	**5105(84.0%)**	**134(78.8%)**	
**≥1**	**971(16.0%)**	**36(21.2%)**	
**Gravidity**			**0.345**
**=0**	**2975(49.0%)**	**77(45.3%)**	
**≥1**	**3101(51.0%)**	**93(54.7%)**	
**Basal FSH (mIU/ml)**	**6.47(5.50,7.70)**	**6.60(5.58,8.09)**	**0.310**
**Tubal factor infertility**			0.004*
**Yes**	**5301(87.2%)**	**161(94.7%)**	
**No**	**775(12.8%)**	**9(5.3%)**	
**Male factor infertility**			0.004*
**Yes**	**410(6.7%)**	**2(1.2%)**	
**No**	**5666(93.3%)**	**168(98.8%)**	
**Endometriosis**			**0.224**
**Yes**	**303(5.0%)**	**5(2.9%)**	
**No**	**5773(95.0%)**	**165(97.1%)**	
**Polycystic ovary syndrome**			0.030*
**Yes**	**589(9.7%)**	**25(14.7%)**	
**No**	**5487(90.3%)**	**145(85.3%)**	
**Diminished ovarian reserve**			**0.498**
**Yes**	**241(4.0%)**	**5(2.9%)**	
**No**	**5835(96.0%)**	**165(97.1%)**	
**IUA**			**0.203**
**Yes**	**97(1.6%)**	**5(2.9%)**	
**No**	**5979(98.4%)**	**165(97.1%)**	
**Scarred uterus**			**0.727**
**Yes**	**285(4.7%)**	**7(4.1%)**	
**No**	**5791(95.3%)**	**163(95.9%)**	
**Insemination**			0.021*
**IVF**	**4582(75.4%)**	**143(84.1%)**	
**IVF + ISCI**	**304(5.0%)**	**8(4.7%)**	
**ICSI**	**1190(19.6%)**	**19(11.2%)**	
**Duration of gonadotrophin (days)**	**11(9,12)**	**10(9,12)**	**0.109**
**Total gonadotrophin dose (IU)**	**2025(1575,2512.5)**	**1875(1425,2362.5)**	0.005*
**No. of embryos transferred**			**0.844**
**=1**	**709(11.7%)**	**19(11.2%)**	
**>1**	**5367(88.3%)**	**151(88.8%)**	
**No. of oocytes retrieved**	**10(8,14)**	**10(8,14)**	**0.982**
**Endometrial thickness (mm)**	**10.90(9.60,12.30)**	**9.80(8.675,11.60)**	< 0.001*
**Endometrial thickness (mm)**			< 0.001*
**<8**	**294(4.8%)**	**27(15.9%)**	
**8-9.9**	**1643(27.0%)**	**60(35.3%)**	
**10-11.9**	**2286(37.6%)**	**47(27.6%)**	
**12-13.9**	**1276(21.0%)**	**23(13.5%)**	
**>14**	**576(9.5%)**	**13(7.6%)**	
**Endometrial thickness (mm)**			< 0.001*
**<8**	**294(4.9%)**	**27(15.9%)**	
**≥8**	**5781(95.1%)**	**143(84.1%)**	
**Endometrial type**			0.030*
**A** ^**a**^	**2725(44.8%)**	**62(36.5%)**	
**B** ^**a,b**^	**3049(50.2%)**	**92(54.1%)**	
**C** ^**b**^	**297(5.0%)**	**16(9.4%)**	
**Endometrial type**			0.008*
**non-C**	**5774(95.0%)**	**154(90.6%)**	
**C**	**297(5.0%)**	**16(9.4%)**	
**Ovarian stimulation protocol**			0.002*
**GnRH agonist protocol**	**4208(69.3%)**	**96(56.5%)**	
**GnRH antagonist protocol**	**901(14.8%)**	**34(20.0%)**	
**Short protocol**	**967(15.9%)**	**40(23.5%)**	

Note: For the quantitative data, the mean ± SD and median (quartile interval) are used to describe the normal distributed data and non-normal distributed data respectively. For categorical data, the number of cases (percentage) is used to describe it. *=statistically significant differences between groups

Compared with IUP patients, patients with EP had significantly thinner EMTs (*P* < 0.001). Furthermore, the prevalence of EP was significantly higher in women with an EMT < 8 mm (8.4%) compared with those with an EMT of ≥ 8 mm (2.4%) (*P* < 0.001). At the same time, those with a type C endometrium were significantly more likely to have EP than those with type A or B endometria (5.1% v 2.2% v 2.9%, *P* = 0.007).

### Univariate regression analysis

The univariate analysis showed that for women with an EMT of < 8 mm, the risk of EP was over three times greater than for those with an EMT ≥ 8 mm (OR 3.713; 95% CI 2.421–5.694; *P <* 0.001). Moreover, the presence of type C endometrium led to a twofold increase in the EP risk compared with non-type C endometria (OR 2.020; 95% CI 1.191–3.424; *P* = 0.009). The results are summarized in Table 2.

**Table 2:** Univariate analysis of factor associated with ectopic pregnancy

**Predictor variable**	Odds **ratio**	95% confidence **interval**	***P value***
**Age (years)**	**0.991**	**0.956–1.027**	**0.621**
**Body mass index (kg/m** ^**2**^ **)**	**0.973**	**0.924–1.025**	**0.301**
**Body mass index (kg/m** ^**2**^ **)**			**0.264**
**<18.5**	**——**	**——**	**——**
**18.5–23.9**	**1.398**	**0.784–2.493**	**0.257**
**>23.9**	**1.076**	**0.465–2.504**	**0.824**
**Infertility type**			
**Primary infertile**	**——**	**——**	**——**
**secondary sterility**	**1.181**	**0.871–1.603**	**0.284**
**Infertility duration (years)**	**0.997**	**0.950–1.047**	**0.912**
**Previous history of ectopic pregnancy**			
**=0**	**——**	**——**	**——**
**≥1**	**1.412**	**0.971–2.054**	**0.071**
**Gravidity**			
**=0**	**——**	**——**	**——**
**≥1**	**0.863**	**0.635–1.172**	**0.346**
**Basal FSH (mIU/ml)**	**1.042**	**0.977–1.112**	**0.210**
**Tubal factor infertility**			
**Yes**	**2.615**	**1.331–5.139**	0.005*
**No**	**——**	**——**	**——**
**Male factor infertility**			
**Yes**	**0.165**	**0.041–0.666**	0.011*
**No**	**——**	**——**	**——**
**Endometriosis**			**0.230**
**Yes**	**0.577**	**0.235–1.416**	
**No**	**——**	**——**	**——**
**Polycystic ovary syndrome**			
**Yes**	**1.606**	**1.042–2.476**	0.032*
**No**	**——**	**——**	**——**
**Diminished ovarian reserve**			
**Yes**	**0.734**	**0.299–1.803**	**0.500**
**No**	**——**	**——**	**——**
**IUA**			
**Yes**	**1.868**	**0.750–4.650**	**0.179**
**No**	**——**	**——**	**——**
**Scarred uterus**			
**Yes**	**0.873**	**0.406–1.877**	**0.727**
**No**	**——**	**——**	**——**
**Insemination**			
**IVF**	**——**	**——**	**——**
**IVF + ICSI**	**0.843**	**0.410–1.735**	**0.643**
**ISCI**	**0.512**	**0.316–0.829**	0.007*
**Source of sperm**			
**husband**	**——**	**——**	**——**
**donation**	**0.000**	**0.000**	**0.996**
**Duration of gonadotrophin (days)**	**0.945**	**0.889–1.005**	**0.072**
**Total gonadotrophin dose (IU)**	**1.000**	**0.999-1.000**	0.003*
**No. of embryos transferred**			
**=1**	**——**	**——**	**——**
**>1**	**0.952**	**0.587–1.545**	**0.844**
**No. of oocytes retrieved**	**0.995**	**0.960–1.030**	**0.758**
**Endometrial thickness (mm)**	**0.81**	**0.747–0.878**	0.000*
**Endometrial thickness (mm)**			< 0.001*
**< 8**	**4.069**	**2.069–8.003**	< 0.001*
**8-9.9**	**1.618**	**0.882–2.969**	0.120*
**10-11.9**	**0.911**	**0.490–1.695**	**0.768**
**12-13.9**	**0.799**	**0.402–1.588**	**0.521**
**> 14**	**——**	**——**	**——**
**Endometrial thickness (mm)**			
**< 8**	**3.713**	**2.421–5.694**	< 0.001*
**≥ 8**	**——**	**——**	**——**
**Endometrial type**			
**A**	**——**	**——**	**——**
**B**	**1.326**	**0.957–1.838**	**0.090**
**C**	**2.368**	**1.349–4.156**	0.003*
**Endometrial type**			
**non C**	**——**	**——**	**——**
**C**	**2.020**	**1.191–3.424**	0.009*
**Ovarian stimulation protocol**			0.002*
**GnRH agonist protocol**	**——**	**——**	**——**
**GnRH antagonist protocol**	**1.654**	**1.111–2.462**	0.013*
**Short protocol**	**1.813**	**1.246–2.639**	0.002*

Note: *=statistically significant differences between groups

### Multivariate stepwise regression analysis

Variables showing *P*-values < 0.1 in the univariate analysis were subsequently included in the multivariate stepwise regression analysis, with the results provided in Table 3.

**Table 3:** Stepwise-Multiple analysis of factors associated with ectopic pregnancy

Predictor variable	Adjusted odds ratio	95% confidence interval	*P* value
**Tubal factor infertility**			
**Yes**	**2.139**	**1.083–4.225**	0.029*
**No**	**——**	**——**	**——**
**Male factor infertility**			
**Yes**	**0.182**	**0.044–0.749**	0.018*
**No**	**——**	**——**	**——**
**Total gonadotrophin dose (IU)**	**0.999** ^**∆**^	**0.999-1.000**	0.008*
**Endometrial thickness (mm)**			
**< 8**	**3.368**	**2.184–5.196**	< 0.001*
**≥ 8**	**——**	**——**	**——**
**Endometrial type**			
**non C**	**1**	**——**	**——**
**C**	**1.897**	**1.110–3.242**	0.019*
**Ovarian stimulation protocol**			0.016*
**GnRH agonist protocol**	**——**	**——**	**——**
**GnRH antagonist protocol**	**1.748**	**1.162–2.628**	0.007*
**Short protocol**	**1.516**	**1.034–2.221**	**0.033**

Note: *=statistically significant differences between groups. ^**∆**^= The specific value of the aOR for the“total gonadotrophin dose”generated by the statistical software range between 0.999 and 1.000. After rounding these values, it became 1.000. Therefore, in the table, we changed the value to 0.999 and marked it with an upward-pointing triangle

After adjusting for confounders, the risk of EP increased more than twofold for women with an EMT of < 8 mm in comparison with those with an EMT of ≥ 8 mm (aOR 3.368; 95% CI 2.184–5.196; *P* < 0.001). Consistent with the univariate analysis, patients with type C endometria still had a nearly twofold risk of having EP (aOR 1.897; 95% CI 1.110–3.242; *P* = 0.019) relative to non-type C endometria.

The use of antagonist protocols (aOR 1.748; 95% CI 1.162–2.628; *P* = 0.007) and TFI (aOR 2.139; 95% CI 1.083–4.225; *P* = 0.029) were also identified as risk factors. On the other hand, a larger dose of total gonadotrophin (aOR 0.999; 95% CI 0.999-1.000; *P* = 0.008) was protective against EP.

## Discussion

To date, this study represents the first and largest retrospective investigation into the influence of combined endometrial features, namely, the endometrium type and EMT, on the risk of EP after fresh embryo transfer. The results indicated that the risk factors for EP were a thin type C endometrium, with TFI, a smaller Gn dose, and the use of a GnRH antagonist protocol being additional risk factors for EP.

Several studies [18–20, 28] have suggested that the presence of a thinner EMT before embryo transfer was more likely to induce EP. However, these studies focused only on freeze-thaw embryo transfer (FET) cycles [18] or confused the use of fresh embryos with FET [19, 20]. Another common feature of previous studies is the absence of information on the endometrial type when investigating EMT. Several researchers have also suggested that the incidence of EP in frozen or donor cycles without ovarian hyperstimulation was lower compared with fresh autologous cycles, thus suggesting that differences in the tubal-uterine environment between cycles contributed to abnormal implantation following embryo transfer [17]. Therefore, the current authors believe that fresh embryo transfer cycles should be analyzed separately.

Consistent with previous studies [6, 7, 9, 18, 19, 26, 29, 30], TFI increased the risk of EP more than twofold. In terms of multiple-embryo transfer, while some studies [18, 20, 26] considered it a risk factor for EP, another [19] suggested that it had no impact. The latter conclusion was supported by the results of the current work, although this could be attributed to the relatively small fraction (728/6246) of the women to whom a single embryo was transferred. Gravidity [18] and previous history of EP [19, 31] represented a similar situation.

Consistent with previous studies, infertility duration [19], method of insemination [18], bFSH [19], BMI [18, 19, 32], infertility type [19], number of oocytes retrieved [19], duration of Gn (days) [19], and age [18, 19] were not found to be risk factors for EP, although one study [20] suggested that age and BMI could independently influence the occurrence of EP. However, unlike the findings of a previous study [19], the current study found a higher Gn dosage to be a protective factor against EP. Furthermore, one study [33] found that the COS cycle was associated with higher uterine peristalsis compared with the natural cycle, with waves mostly moving from the cervix to the fundus. Even at a high level of progesterone, the intensity of the waves did not decrease to natural levels before embryo transfer. Thus, it is evident that COS influences endometrial motility, although the relationship between the Gn dosage and the frequency/direction of endometrial peristalsis wave is yet to be studied.

The risk of EP was found to be higher in when using the GnRH antagonist protocol compared with the GnRH agonist protocol. This result is consistent with previous studies [17, 20], indicating that the incidence of EP can be influenced by the selected protocol.

The present study showed that an EMT of < 8 mm increased the risk of EP more than twofold in comparison with an EMT of ≥ 8 mm. Previous studies [18, 19] have shown a link between thinner EMT and higher EP risk, with one study [18] even reporting an increased EP risk with an EMT of < 12 mm in the frozen embryo cycle. Similarly, a different investigation showed that EP patients had thinner EMTs after a positive pregnancy test [34].

It remains unclear why patients with a thin EMT have a higher risk of EP. This could partly be due to an abnormally high oxygen tension which is known to be detrimental to embryonic development due to the production of reactive oxygen species [35–37]. As published by Bartelmez [35], the blood vessels in the endometrium’s basal layer are large spiral arteries, while those in the functional layer are capillaries. Thus, unlike the usual low oxygen tension seen at the endometrial surface, the high oxygen content close to the basal layer could create unfavorable conditions for embryo development. This is because a thin EMT causes the embryo to be much closer to the basal endometrium layer’s spiral arteries, exposing it to higher concentrations of reactive oxygen species. However, in this case, the fallopian tube’s lower oxygen concentration could be more suitable for embryo implantation [38].

Another possible mechanism that could link EP to thin EMT is uterine peristalsis. It has been reported that compared with IUP, the uterine peristaltic wave frequency is increased in patients with EP, although the differences were not significant [39] due to the uneven distribution of the sample size. Patients with a thicker EMT were also reported to have an increased risk of placenta previa [40], and as such, the authors postulated that higher EMT was associated with uterine peristalsis that affected embryo implantation sites. Although the results of the above two studies appear contradictory, it should be noted that neither considered the type and direction of the uterine peristaltic wave, as both factors are important in the assessment of uterine activity [33, 41]. Indeed, embryos can be removed from their original transfer site [42] if the peristaltic waves occur from the fundus to the cervix [33, 39, 43], resulting in the movement of a transferred embryo in an inferior direction that increases the possibility of placenta previa. In contrast, if the waves are in the opposite direction, this may increase the likelihood of EP [39, 44, 45].

A third possible explanation could be endometrial receptivity. Many studies have shown that EMT is associated with uterine receptivity [46–51]. In fact, compared with endometria of normal thickness, thin endometria area reported to contain different amounts of cytokines. At the same time, many genes which are related to anti-oxidative stress and metabolism were found to be down-regulated [52]. Currently, there are many criteria for diagnosing endometrial receptivity, including several molecular diagnostic models [53], and thus the associations between EMT characteristics and endometrial receptivity require further investigation.

The other arguably most important finding of the current study was that a type C endometrium increased the risk of EP nearly twofold. The hyperechoic middle line is usually assumed to represent the uterine cavity, with the other two considered to be the junction between the endometrium and myometrium. However, the mechanism of hyperechoic endometrium imaging remains disputable. In this context, Fleischer et al. [54] suggested that, during the late secretory phase, a homogeneous hyperechoic endometrium could indicate a stromal edema [55], referred to as endometrial decidualization when sex hormones cause the endometrium to change from the proliferative phase to a secretory one. The absence of decidualization is related to infertility and recurrent spontaneous abortion (RSA). Some researchers further believe that a premature endometrium secretory pattern is bad for pregnancy [56], while Friedler [57] considered that the type of endometrium could provide valuable predictive information regarding pregnancy. Based on the above, the current authors believe that a type C endometrium could be related to a shift in the embryo implantation window due to early endometrial transformation. Therefore, patients with a type-C endometrium, along with an EMT of < 8 mm on trigger day should be adequately counseled, and their endometrial peristaltic wave/receptivity should also be examined before transfer.

After COS, patients tend to have more frequent uterine peristaltic waves than during natural cycles [33]. However, to date, no study has reported an association between EMT and peristaltic wave frequency, nor between other indicators such as endometrium type and peristaltic wave direction.

The main strength of this work is that, for the first time, the influence of both endometrium type and EMT on EP frequency was determined. Furthermore, unlike previous studies, fresh cycles were studied separately from freeze-thaw transfers. Finally, several previous studies included HP in the EP group even though in HP, one of the embryos is normally implanted in the uterine cavity. Since endometrial characteristics affect EP occurrence, HP should be excluded.

Furthermore, this single study not only included a large sample size but both the EMT and endometrium type were assessed by the same trained sonographers. Similarly, the same trained embryologists cultured and evaluated all embryos. These practices reduced inter-observer and inter-center variations. Finally, almost all the embryos which were transferred during the fresh embryo cycles were of good quality (the fragmentation rates were < 20%) at the cleavage stage on day 3 in our reproductive center. This helped to overcome the influence of confounding factors such as the stage of embryonic development and embryo quality.

However, due to its retrospective design, this study was not without limitations. Confounders which are known [58] to affect pregnancy outcomes, namely, alcohol consumption, smoking, and risky sexual behaviors, were not included, as factors such as smoking and alcohol which could have negatively influenced pregnancy were consciously reduced by couples seeking IVF, thus these factors can be considered to have had little impact.

## Conclusions

A type-C endometrium and a thin EMT were linked to a significantly higher risk of EP. Combining information on endometrium type and EMT could be a quantitative marker of uterine peristalsis or even of endometrial receptivity in fresh embryo cycles. Furthermore, patients at high risk of EP, especially if diagnosed with TFI and a thin EMT combined with a type C endometrium, should be further evaluated to determine whether the ET procedure should be carried forward. Finally, prior to ET, EP monitoring can be strengthened for high-risk groups, probably through endometrial peristaltic wave/receptivity examination, and this could be supplemented with medical treatments [59, 60] for controlling peristaltic waves to further prevent EP in high-risk populations.

## Data Availability

The datasets used and/or analyzed during the current study are available from the corresponding author on reasonable request.
